# Relevance of Serum Leptin and Leptin-Receptor Concentrations in Critically Ill Patients

**DOI:** 10.1155/2010/473540

**Published:** 2010-09-07

**Authors:** Alexander Koch, Ralf Weiskirchen, Henning W. Zimmermann, Edouard Sanson, Christian Trautwein, Frank Tacke

**Affiliations:** ^1^Department of Medicine III, RWTH-University Hospital Aachen, Pauwelsstraße 30, 52074 Aachen, Germany; ^2^Institute of Clinical Chemistry and Pathobiochemistry, RWTH-University Hospital Aachen, Pauwelsstraße 30, 52074 Aachen, Germany

## Abstract

The adipocyte-derived cytokine leptin was implicated to link inflammation and metabolic alterations. We investigated the potential role of leptin components in critically ill patients, because systemic inflammation, insulin resistance, and hyperglycemia are common features of critical illness. Upon admission to Medical Intensive Care Unit (ICU), free leptin and soluble leptin-receptor serum concentrations were determined in 137 critically ill patients (95 with sepsis, 42 without sepsis) and 26 healthy controls. Serum leptin or leptin-receptor did not differ between patients or controls and were independent of sepsis. However, serum leptin was closely associated with obesity and diabetes and clearly correlated with markers of metabolism and liver function. Leptin-receptor was an unfavourable prognostic indicator, associated with mortality during three years follow-up. Our study indicates a functional role of leptin in the pathogenesis of severe illness and emphasizes the impact of complex metabolic alterations on the clinical outcome of critically ill patients.

## 1. Introduction

Hyperglycemia, glucose intolerance, and insulin resistance are common features of critically ill patients, especially in patients with sepsis or septic shock, even in those without preexisting diabetes mellitus [1–3]. In patients with obesity, metabolic syndrome, and type 2 diabetes, several adipocytokines have been identified that mediate agonistic and antagonistic effects on insulin resistance [4, 5]. A link between adipocytokines, inflammation, and systemic insulin resistance has been established in obese and diabetic patients [5]. In critically ill patients, little is known about the actions of the different adipokines, especially about their potential impact on insulin resistance.

Since its identification in 1994 leptin, a 16-kilodalton hormone, has been investigated for its role in signalling food intake, glucose homeostasis, and energy expenditure through hypothalamic pathways [6–8]. Circulating leptin levels directly reflect adipose tissue mass and recent nutritional status in noncritically ill individuals [9]. The mechanisms of leptin expression are unclear, possibly insulin-stimulated glucose metabolism and peroxysome proliferator-activated receptor gamma (PPAR*γ*) are involved in adipocyte leptin induction [10, 11]. Leptin exerts its various actions on glucose metabolism and energy expenditure via binding to the leptin-receptor in the brain and peripheral tissues as pancreas, liver, adipose tissue, and in the immune system [12]. In clinical settings, free-circulating leptin as well as soluble leptin-receptors that form complexes with circulating leptin are used to understand the pathogenetic role of leptin in regulating the inflammatory-metabolic response [13]. Various animal and human studies have shown that administration of endotoxin, TNF*α*, and other cytokines as inducers of severe systemic inflammation result in a significant elevation of serum leptin concentrations [14, 15].

Our study investigated serum leptin and leptin-receptor serum concentrations in a large cohort of critically ill patients (septic and nonseptic patients) from a medical ICU in order to understand the potential involvement of leptin and leptin-receptor in the pathogenesis of insulin resistance in critical illness, its regulation in severe systemic inflammation, and its potential clinical use as a biomarker in ICU patients.

## 2. Patients and Methods

### 2.1. Study Design and Patient Characteristics

The study was approved by the local ethics committee. Written informed consent was obtained from the patient, his or her spouse, or the appointed legal guardian. A total of 137 patients (85 male, 52 female with a median age of 63 years; range 18–81 years) was studied (Table 1). Patients were included consecutively upon admission to the ICU, if they were admitted to the Medical ICU of the University Hospital Aachen due to critical illness. Patients were not included in this study, if they were expected to have a short-term (<72 hours) intensive care treatment due to postinterventional observation or acute intoxication. Patient data, clinical information, and blood samples were collected prospectively.

The control group consisted of 26 healthy nondiabetic blood donors (17 male, 9 female, with a median age of 56 years; range 24–66 years) with normal values for blood counts, C-reactive protein, and liver enzymes.

### 2.2. Characteristics of Sepsis and Nonsepsis Patients

Among the 137 critically ill patients enrolled in this study, 95 patients conformed to the criteria of bacterial sepsis, according to the criteria of the American College of Chest Physicians & the Society of Critical Care Medicine Consensus Conference Committee for severe sepsis and septic shock [16]. In the majority of sepsis patients, the identified origin of infection was pneumonia (*n* = 54). Non-sepsis patients did not differ in age or sex from sepsis patients and were admitted to the ICU due to cardiopulmonary disorders (myocardial infarction, pulmonary embolism, and cardiac pulmonary edema; *n* = 17), decompensated liver cirrhosis (*n* = 14), or other critical conditions (*n* = 11). In sepsis patients, significantly higher levels of laboratory indicators of inflammation (i.e., C-reactive protein, procalcitonin, white blood cell count) were found than in non-sepsis patients (Table 1, and data not shown). Nevertheless, both groups did not differ in APACHE II score, vasopressor demand, or laboratory parameters indicating liver or renal dysfunction (data not shown). Among all critical care patients, 29.9% died at the ICU and 51.8% of the total initial cohort died during the overall follow-up of 900 days (Table 1). In sepsis and non-sepsis patients, no significant differences in rates of death and survival were observed (data not shown).

### 2.3. Comparative Variables

The patients in the sepsis and non-sepsis groups were compared by age, sex, body mass index (BMI), preexisting diabetes mellitus, and severity of disease using the APACHE II score at admittance. Intensive care treatment like volume therapy, vasopressor infusions, demand of ventilation and ventilation hours, antibiotic and antimycotic therapy, renal replacement therapy, and nutrition were recorded, alongside a large number of laboratory parameters that were routinely assessed during intensive care treatment.

### 2.4. Quantification of Human Leptin and Leptin-Receptor

Human leptin serum concentrations were determined with a commercial ELISA (Cat. No. RD191001100, Bio Vendor). Intraassay (interassay) coefficient of variation (CV) ranged from 4.2% to 7.6% (*n* = 8) (4.4%–6.7% (*n* = 6)). Human leptin-receptor concentrations in serum were determined using a commercially available ELISA (Cat. No. RD194002100, Bio Vendor, Candler, NC). Intraassay (interassay) coefficient of variation (CV) ranged from 7.1% to 7.3% (*n* = 8) (6.2%–9.8% (*n* = 6)).

### 2.5. Statistical Analysis

Due to the skewed distribution of most of the parameters, data are given as median, minimum, maximum, 95% confidence interval. Differences between two groups are assessed by Mann-Whitney-*U*-test and multiple comparisons between more than two groups have been conducted by Kruskal-Wallis-ANOVA and Mann-Whitney-*U*-test for post hoc analysis. Box plot graphics illustrate comparisons between subgroups. They display a statistical summary of the median, quartiles, range, and extreme values. The whiskers extend from the minimum to the maximum value excluding outside and far out values which are displayed as separate points. An outside value (indicated by an open circle) is defined as a value that is smaller than the lower quartile minus 1.5-times interquartile range, or larger than the upper quartile plus 1.5-times the interquartile range. A far out value is defined as a value that is smaller than the lower quartile minus three times interquartile range, or larger than the upper quartile plus three times the interquartile range. All values, including “outliers”, have been included for statistical analyses. Correlations between variables have been analysed using the Spearman correlation tests, where values of *P* < .05 were considered statistically significant. The prognostic value of the variables was tested by univariate and multivariate analysis in the Cox regression model. Kaplan Meier curves were plotted to display the impact on survival. All statistical analyses were performed with SPSS version 12.0 (SPSS, Chicago, IL, USA).

## 3. Results

### 3.1. Leptin and Leptin-Receptor Do Not Differ in Patients with Critical Illness from Healthy Controls

**Figure 1:**
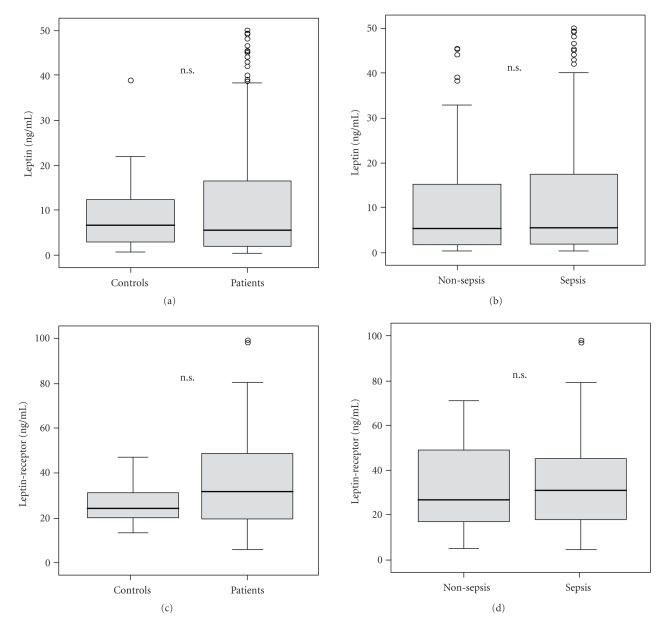
Serum leptin and leptin-receptor concentrations in critically ill patients. (a) Serum leptin levels are not different in critically ill patients (*n* = 137, median 5.5 ng/mL, range 0.4–49.6) as compared to healthy controls (*n* = 26, median 6.6 ng/mL, range 0.7–38.6). (b) No significant differences are detected between ICU patients with sepsis (*n* = 95, median 5.5 ng/mL, range 0.4–49.6) and nonseptic etiology (*n* = 42, range 5.4 ng/mL, range 0.4–45.1) of critical illness. (c) Leptin-receptor serum concentrations do not differ between critically ill patients (median 31.5 ng/mL, range 5.7–126.9) and healthy controls (median 23.9 ng/mL, range 13.1–46.4). (d) No difference in serum leptin-receptor concentrations can be observed between patients with (median 31.8 ng/mL, range 5.7–126.9) or without sepsis (median 29.0 ng/mL, range 7.4–124.7) at admission to the ICU. Box plot are displayed, where the bold line indicates the median per group, the box represents 50% of the values, and horizontal lines show minimum and maximum values of the calculated nonoutlier values; asterixes and open circles indicate outlier values.

It had been reported that circulating leptin levels were low in critically ill patients upon admission to the ICU, possibly due to an acute stress response, with lowest levels in patients with sepsis [17]. We therefore tested free leptin and soluble leptin-receptor serum concentrations in 137 critically ill patients upon admission to our Medical ICU. Surprisingly, we did not observe significant differences in ICU patients as compared to healthy controls (Figure 1). Moreover, when we compared patients with sepsis (*n* = 95) and patients without sepsis (*n* = 42), no significant difference for either leptin or leptin-receptor concentrations could be detected (Figures 1(b) and 1(d)). Additionally, we performed subgroup analyses comparing patients with sepsis of pulmonary origin to those with abdominal or other septic focuses. However, leptin and leptin-receptor serum concentrations did not differ between these subgroups (data not shown).

### 3.2. Leptin and Leptin-Receptor Are Associated with Obesity and Diabetes in Critically Ill Patients

**Figure 2:**
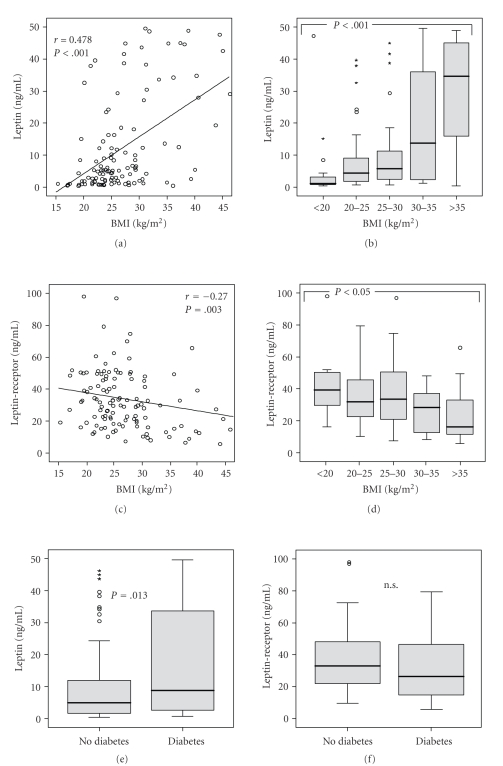
Association of serum leptin and leptin-receptor with obesity and diabetes in critically ill patients. (a) Serum leptin concentrations in ICU patients are correlated with the body-mass index (BMI). Spearman rank correlation test, correlation coefficient *r*, and *P*-values are given. (b) Among critically ill patients, serum leptin levels are significantly associated with the patient's BMI. Box-plot graphics are displayed for different classes of BMI; Kruskal-Wallis-test is used to assess significance of the differences. (c, d) In line, an inverse association between patient's BMI or obesity and serum leptin-receptor levels can be detected. (e) Serum leptin is significantly elevated in critically ill patients with preexisting diabetes in comparison to patients without diabetes. (f) Serum leptin-receptor concentrations do not differ in critically ill patients with or without diabetes. *P*-values (*U*-test) are given in the figure. Box plot are displayed, where the bold line indicates the median per group, the box represents 50% of the values, and horizontal lines show minimum and maximum values of the calculated nonoutlier values; asterixes and open circles indicate outlier values.

Leptin is a cytokine mainly derived from adipose tissue and its concentrations directly reflect adipose tissue mass and the current nutritional status in noncritically ill individuals [4]. Consequently, we observed a close correlation between the patients' body-mass indices (BMI) and their leptin serum concentrations (*r* = 0.478, *P* < .001, Spearman rank correlation test; Figure 2(a)). By grouping the patient cohort to different BMI classes (<20, 20–25, 25–30, 30–35, >35), a clear increase in serum leptin with the BMI can be demonstrated (Figure 2(b)). In line, an inverse association between BMI and the circulating leptin-receptor could be revealed, with lowest leptin-receptor concentrations in obese patients with a BMI >35 kg/m² (Figures 2(c) and 2(d)).

Patients with a preexisting diabetes (*n* = 46) had significantly higher serum leptin levels (median 8.8 ng/mL) than patients without diabetes (*n* = 91, median leptin 4.9 ng/mL, *P* = .013; Figure 2(e)). However, leptin-receptor serum concentrations did not differ in critically ill patients with or without diabetes (Figure 2(f)).

### 3.3. Leptin Is Correlated to Procalcitonin and Liver Dysfunction in Critical Illness

Leptin has been functionally linked to inflammatory proteins as it may directly interact with C-reactive protein [18]. We observed an inverse correlation between leptin and procalcitonin, but not to C-reactive protein or interleukin-6 (Table 2). With respect to organ dysfunction in the critically ill patients, we could not detect a significant correlation between leptin and renal function (e.g., glomerular filtration rate, cystatin C, creatinine; data not shown). However, the hepatic biosynthetic capacity was closely associated with serum leptin concentrations, as evidenced by significant correlations to serum protein, albumin, prothrombin time, antithrombin III, and pseudocholinesterase activity (Table 2).

### 3.4. Leptin Is Associated with Glucose and Lipid Metabolism

Several observations in mouse models suggested that the actions of leptin on glucose homeostasis are independent of its effects on food intake, but associated with proper function of hypothalamic leptin-receptors [19, 20]. We observed a close correlation of insulin and C-peptide with leptin and an inverse correlation with leptin-receptor (Table 2). Furthermore, markers of lipid metabolism, for example, total cholesterol, HDL cholesterol, and LDL cholesterol were closely associated with leptin serum concentrations (Table 2).

### 3.5. Leptin-Receptor Is Not Linked to Metabolic Parameters, but to Procalcitonin and Parameters Reflecting Cholestasis

In contrast to leptin, a correlation between serum leptin-receptor concentrations and metabolic parameters could not be found (Table 2). An association between leptin-receptor and procalcitonin as well as insulin could be observed, but not with other inflammatory or metabolic parameters (Table 2, and data not shown). Interestingly, leptin-receptor showed significant correlations to various parameters reflecting cholestasis such as bilirubin, conjugated bilirubin, gamma-glutamyltranspeptidase, and alkaline phosphatase activity (Table 2). This might indicate that biliary excretion could be possibly involved in leptin-receptor clearance.

### 3.6. High Leptin-Receptor Levels Indicate Unfavourable Prognosis in Critically Ill Patients

**Figure 3:**
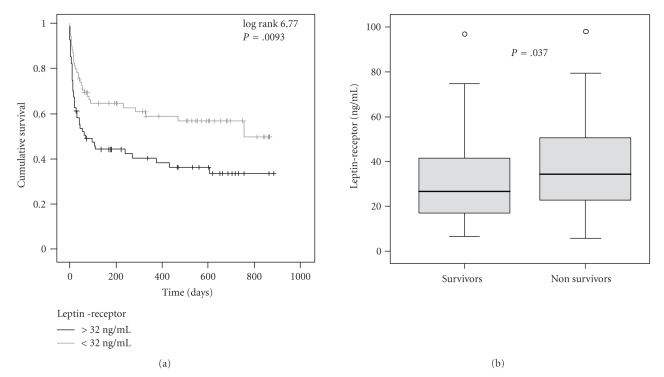
Prognostic relevance of serum leptin-receptor in critically ill patients. (a) Kaplan-Meier survival curves of ICU patients (*n* = 137) are displayed, showing that patients with high leptin-receptor levels (>32 ng/m/L, black) have an increased mortality as compared to patients with low serum leptin-receptor (≤32 ng/mL, grey). *P*-value from Cox regression analysis is  .034. (b) Patients that die during follow-up period treatment (*n* = 71, 51.8%) have significantly (*P* = .037) higher serum leptin-receptor levels on admittance to ICU than survivors (*n* = 66, 48.2%). Box plot are displayed, where the dotted line indicates the median per group, the box represents 50% of the values, and horizontal lines show minimum and maximum values of the calculated nonoutlier values; asterixes and open circles indicate outlier values.

Cox regression analyses and Kaplan-Meier curves were used to assess the impact of leptin and leptin-receptor on ICU and overall survival during a nearly three-year follow-up among critically ill patients. No significant association between leptin and the ICU- or overall-survival of the patients could be identified using uni- and multivariate Cox regression analysis (data not shown). Remarkably, leptin-receptor serum concentrations upon admission to the Medical ICU were an unfavourable indicator of ICU survival (*P* = .047) as well as of overall survival (*P* = .034, Cox regression analysis). Using a cut-off value for leptin-receptor of 32 ng/mL, Kaplan-Meier curves were plotted to display mortality (log rank 6.77; Figure 3(a)). In line, surviving patients had significantly lower leptin-receptor concentrations (median 26.8 ng/mL) than nonsurvivors (median 34.5 ng/mL, *P* = .037, *U*-test; Figure 3(b)).

## 4. Discussion

Hyperglycemia and insulin resistance are common in critically ill patients and have been identified as adverse prognostic predictors in ICU patients. In obesity and type 2 diabetes, adipocytokines are critical mediators linking chronic inflammatory conditions to systemic insulin resistance [5]. The role of adipocytokines in sepsis and nonseptic ICU patients is currently unclear. The best studied adipocytokine in critical illness at present is resistin, which was suggested to serve as an acute-phase component as it is strongly upregulated in patients with severe sepsis and septic shock [21, 22]. Very little is known about the potential role of leptin in this clinical condition.

In a recently published study, it has been reported that serum leptin concentrations are low in all critically ill patients on admission to the ICU, with lowest levels in sepsis patients [17]. As a possible mechanism, reduced synthesis or increased removal either by extravasation due to capillary leakage in sepsis or increased metabolic clearance has been speculated [17]. In this study by Langouche et al., the SOFA score on admission was statistically significantly higher in sepsis patients as compared to nonseptic patients. Moreover, a prior study demonstrated decreased plasma leptin levels due to trauma which were explained to be partly related to the initial fasting conditions, because refeeding elevated serum leptin concentrations to normal levels [23].

In our study, the leptin and leptin-receptor concentrations did not differ in patients with critical illness from healthy controls. Subgroup analysis revealed no significant difference in sepsis and non-sepsis patients as well (Figure 1). This difference might very likely be related to our cohorts of septic and nonseptic patients, which were very homogenous in terms of severity of illness as reflected by APACHE II and SOFA score. Moreover, our study population had a slightly higher median BMI and therefore possibly a better nutritional status in the subgroups of patients with and without proven sepsis. However, in contrast to prior observations, our data indicate that circulating leptin and leptin-receptor levels are not severely dysregulated in critically ill patients at the point of admission to the ICU.

Leptin has various peripheral and central targets, including cells of the brain, pancreas, liver, adipose tissue, and immune system [12]. In animal models, deletion of the cerebral leptin-receptor leads to obesity and elevated plasma levels of leptin, glucose, and insulin [24]. Leptin deficiency in humans either due to an absolute shortage of leptin or due to leptin-receptor mutations causes severe early onset obesity, hyperphagia, hyperinsulinemia, hypogonadism, and impaired T cell function [25]. In individuals with leptin-receptor defects, the features of leptin deficiency appear ameliorated, which might give a hint for the existence of alternative, leptin-receptor independent, leptin signalling pathways [26]. In line with the current view of leptin as being a cytokine, mainly derived from adipose tissue and reflects adipose tissue mass, we found a close association to the patients' BMI (Figures 2(a) and 2(b)). Accordingly, an inverse association could be observed for the circulating leptin-receptor (Figures 2(c) and 2(d)), which is expressed by various tissues and serves as a binding partner for leptin in the circulation [24].

The regulation of glucose metabolism by leptin is likely independent of its effect on food intake and energy expenditure. Hypothalamic expression of leptin-receptor in animal model resulted in modest reduction of food intake and body fat mass, but in normalized blood glucose and insulin levels [19]. The present data suggest the existence of two leptin signalling pathways: the leptin-receptor mediated JAK-STAT (Janus kinase signal transducers and activators of transcription) signalling for regulation of food intake and body weight, and PI3K (phosphoinositide-3 kinase) pathway for regulation of glucose metabolism [19, 20, 27]. These experimental findings might very well explain why we did not only observe a strong association between patients' BMI and leptin, but also between glucose metabolism (e.g., insulin secretion) and serum leptin levels in critically ill patients.

The possible interaction between leptin and systemic inflammation is a matter of ongoing debate. It has been reported that human C-reactive protein (CRP) inhibits the binding of leptin to its specific receptor and blocks signal transduction in cultured cells and mouse models; thus, it might attenuate the physiological function of leptin and contribute to the concept of “leptin resistance” [18]. In our study, we could demonstrate an inverse correlation between leptin and procalcitonin and a direct correlation between leptin-receptor and procalcitonin, but not with “classical” markers of inflammation as C-reactive protein or interleukin-6. Possibly, acute bacterial inflammation, as displayed by procalcitonin, in critically ill patients contributes to the state of “leptin resistance,” yet the clinical impact of “leptin resistance” in critical illness is still unsettled.

Furthermore, we analyzed the association of leptin with markers of organ function in ICU patients. Leptin did not correlate with renal function as reflected by glomerular filtration rate, cystatin C, and creatinine. We observed that the hepatic biosynthetic capacity was closely related to leptin levels (Table 2). This finding in critically ill patients is in contrast to reports from patients with liver cirrhosis, in which leptin levels are rather upregulated [13, 28].

Remarkably, leptin-receptor serum concentrations upon admission to the Medical ICU were an unfavourable indicator of ICU survival (*P* = .047) as well as of overall survival (*P* = .034) (Figures 3(a) and 3(b)). Surviving patients had significantly lower leptin-receptor concentrations than nonsurvivors. This observation was highly unexpected, especially since leptin-receptor was not an obvious surrogate marker for other alterations, for example, inflammation, organ failure, in critically ill patients. The association with survival was independent from other parameters by multivariate regression analysis (data not shown). The exact functional contribution of soluble leptin-receptor or free leptin in the pathogenesis of critical illness or sepsis is currently unclear, and additional studies in experimental models are warranted. It is also important to note that future studies should incorporate longitudinal measurements of leptin and leptin receptor during the course of critical illness, as this might help to clarify the potential pathogenic contributions at different phases of initiation and recovery from acute illness. However, the association of leptin-receptor with survival raises the possibility that including serum leptin-receptor concentrations might improve the validity of prognostic assessments in critically ill patients upon admission to the ICU.

## 5. Conclusions

Although leptin and leptin-receptor serum concentrations do not differ in patients with critical illness or in the subgroups of patients with and without sepsis from healthy controls, serum leptin in critically ill patients is closely correlated with the patients' BMI and metabolic alterations. The possible functional role of leptin in the pathogenesis of severe illness warrants further studies. However, soluble leptin-receptor turned out to be an independent prognostic marker at admission to the Medical ICU, thereby emphasizing the impact of the complex metabolic alterations on the clinical outcome of critically ill patients.

## Figures and Tables

**Table 1:** Characteristics of the study population.

Parameter	All ICU patients	Sepsis	Nonsepsis
Number	137	95	42
Sex (number male/number female)	85/52	59/36	26/16
Sex (% male/female)	62/38	62/38	62/38
Age median (years) (range)	63 (18–81)	64 (20–81)	60 (18–79)
BMI median (kg/m²) (range)	25.4 (15.3–59.5)	25.65 (15.3–59.5)	24.9 (17.5–37.4)
Days at ICU median (range)	8 (1–137)	10 (1–137)	6 (1–45)
Days in hospital median (range)	25 (2–151)	30 (2–151)	14 (2–85)
Death at ICU *n* (%)	41 (29.9)	30 (31.6)	11 (26.2)
Death during follow-up *n* (%)	71 (51.8)	49 (51.6)	22 (52.4)
C-reactive protein median (mg/dL) (range)	112 (5–230)	167 (5–230)	14.5 (5–164)
Procalcitonin median (*μ*g/L) (range)	0.9 (0.1–207.5)	2.2 (0.1–207.5)	0.2 (0.1–36.5)
IL-6 median (ng/L) (range)	110 (2–1000)	170 (7.7–1000)	40.5 (2–1000)
IL-10 median (ng/L) (range)	16 (5–1500)	20 (5–1500)	5.9 (5–750)
Protein median (g/L) (range)	52.5 (21–77)	52 (21–77)	55.5 (31–73)
Prothrombin time median (%) (range)	73 (11–100)	75 (11–100)	69 (13–100)
Creatinine median (mg/dL) (range)	1.6 (0.1–13.1)	1.9 (0.1–10.7)	1.2 (0.3–13.1)
Cystatin C median (mg/L) (range)	1.76 (0.41–7.30)	1.89 (0.41–6.33)	1.34 (0.41–7.30)
Glucose median (mg/dL) (range)	134 (47–663)	126 (47–299)	155 (65–663)
Insulin median (mU/L) (range)	9.8 (0.2–1000)	7.7 (0.2–438.0)	25.0 (0.2–1000)
C-peptide median (nmol/L) (range)	1.66 (0–13.0)	1.56 (0–13.0)	2.01 (0–11.6)
APACHE II score median (range)	14 (0–31)	14 (0–31)	15 (0–28)
SAPS-2 score median (range)	43 (0–80)	43 (0–79)	41.5 (13–80)

BMI: body mass index; IL: interleukin; ICU: intensive care unit; APACHE: Acute Physiology and Chronic Health Evaluation.

**Table 2:** Correlation analysis.

	Leptin		Leptin-receptor	
	*r*	*P*	*r*	*P*
*Inflammation*				
Procalcitonin	−0.179	.043	0.252	.004
Interleukin-10	−0.190	.049	—	n.s.

*Liver synthesis capacity*				
Protein concentration	0.342	<.001	—	n.s.
Albumin concentration	0.295	.001	—	n.s.
Pseudocholinesterase	0.281	.001	−0.217	.012
Prothrombin time (%)	0.266	.002	—	n.s.
Antithrombin III	0.234	.012	−0.22	.017

*Cholestasis*				
*γ*GT	—	n.s.	0.204	.017
AP	—	n.s.	0.345	<.001
Bilirubin (total)	—	n.s.	0.298	<.001
Bilirubin (conjugated)	—	n.s.	0.373	<.001

*Glucose metabolism*				
Insulin	0.430	<.001	−0.294	.001
C-peptide	0.285	.001	—	n.s.

*Lipid metabolism*				
Cholesterol	0.216	.013	—	n.s.
HDL cholesterol	0.269	.003	—	n.s.
LDL cholesterol	0.270	.003	—	n.s.

*Adipocytokines*				
Adiponectin	−0.223	.015	0.548	<.001
Leptin	—	—	−0.587	<.001

*γ*GT: gamma-glutamyltranspeptidase; AP: alkaline phosphatase; HDL: high-density lipoprotein; LDL: low-density lipoprotein.
